# Editorial: Developmental anomalies in the lung and their impact on later life

**DOI:** 10.3389/fped.2023.1302593

**Published:** 2023-10-10

**Authors:** Theodore Dassios

**Affiliations:** ^1^ Women and Children’s Health, School of Life Course Sciences, Faculty of Life Sciences and Medicine, King’s College, London, United Kingdom; ^2^ Neonatal Intensive Care Unit, University of Patras, Patras, Greece

**Keywords:** prematurity, primary ciliary dyskinesia, exercise capacity, mechanical ventilalion, pulmonary artery agenesis

**Editorial on the Research Topic** Developmental anomalies in the lung and their impact on later life

Lung development is a complex process that begins prenatally and continues until young adulthood. The normal course of lung development can be affected by various parameters such as prematurity, congenital disorders and environmental factors which may have a long lasting effect on lung structure and function. The aim of this research topic was to describe new approaches in the assessment of lung development after birth, considering morphological and functional aspects. It also aimed to highlight recent advances in the monitoring of lung development in health and disease during childhood, and describe consequences of developmental defects on lung function in later life.

Premature infants are often ventilated in the early postnatal period and an effort is made to minimize iatrogenic lung injury by using appropriate lung volumes, which are adequate for gas exchange but do not excessively large that could cause overdistention and lung damage. Dassios et al. measured the slopes of volumetric capnography and the ventilation to perfusion ratio in 25 extremely preterm infants to test the hypothesis that gas exchange in ventilated preterm infants occurs both at the level of the alveoli and via mixing of fresh dead space gas in the airways. They concluded that abnormal gas exchange was associated with lung disease at the alveolar level and found no evidence of gas exchange impairment originating at the level of the airways. The authors confirmed that targeted tidal volumes at values below the physiological dead space cannot be associated with efficient gas exchange (Dassios et al.).

While the immediate impact of mechanical ventilation on lung function is undoubtedly injurious, the long term effect of invasive support is also an area of growing research interest in the recent years. Di Filippo et al. aimed to assess the effect of mechanical ventilation on lung function in a group of very preterm-born children studied at 11 years of age. The authors performed a comprehensive respiratory evaluation in 55 children which included spirometry, measurement of lung volumes, lung diffusion capacity and measurement of the fractional exhaled nitric oxide. They reported that there was no difference in any of these outcomes between ventilated and unventilated children. Their cohort included children with a mean gestational age of 30.6 weeks and a mean duration of invasive ventilation of one day, suggesting that such a brief course of invasive ventilation in this population of relatively mature preterms was not associated with a long-lasting impairment in lung function (Di Filippo et al.).

Lung function testing, however, might fail to capture the fine granularity of the spectrum of lung disease following premature birth, which might become apparent only under conditions of increased cardio-respiratory demand such as during moderate to intense aerobic exercise. O’Dea et al. reviewed the literature on the long-term cardiopulmonary outcomes following preterm birth during the current surfactant era, and aimed to outline the current knowledge of cardiopulmonary exercise testing in the assessment of children born preterm. They described that preterm-born children have increased respiratory symptoms and disrupted lung development with significant structural and functional lung sequelae. The authors highlighted that expiratory flow limitation and an altered ventilatory response consisting of rapid, shallow breathing were observed during exercise. The association, however, between exercise capacity and the traditional resting respiratory assessments was not clear. Some constraints such as the heterogeneity of study participants, treatments and exercise protocols, precluded our understanding of exercise capacity limitations in children born preterm. The authors suggested that the role of exercise interventions in mitigating the risk of chronic lung disease should be a focus of future randomised controlled trials (O’Dea et al.).

Other than prematurity, congenital defects can also impact on lung development with long lasting effects. Miseviciene et al. described a novel case of a rare combination of unilateral pulmonary artery agenesis and Kommerell's diverticulum which puts affected individuals at a high risk for pulmonary hypertension and rupture of the diverticulum. The authors described how a one-year old girl presented with prolonged cough and wheezing and a hypoplastic left lung in a chest radiograph. She later underwent echocardiography and chest computed tomography which confirmed an absence of the left pulmonary artery and right arch of the aorta and an anomaly of the subclavian arteries. She was referred for ongoing monitoring for possible development of pulmonary hypertension and compression from the vascular structures to the airways which might require surgical intervention (Miseviciene et al.).

Primary ciliary dyskinesia might affect lung development with severe lifelong implications but the disease is closely monitored epidemiologically predominantly in North America and Europe. The incidence of the disease can thus be underestimated particularly in developing countries, due to a lack of awareness and diagnostic facilities. Castillo et al. performed a mini-review which aimed to highlight the lack of standardized diagnostic and treatment guidelines for the disease in Latin America and compared North American and European diagnosis and management recommendations. They highlighted that certain diagnostic tools and treatment options are not universally accessible in Latin America and identified fifteen articles that provided recommendations on respiratory management, a minority of whom originated from Latin America. The authors commented on the relative absence of documentation, research, and recommendations regarding the prevalence of the disease in Latin America, likely due to unfavorable economic conditions. They suggested that in developing countries, the PICADAR score, which is based on clinical characteristics, can serve as an alternative method to identify patients who require further testing and have a higher probability of a diagnosis of the disease (Castillo et al.).

In conclusion, prematurity, congenital disease and socioeconomic disparities can affect the provision of care in developmental respiratory disease. These conditions could be focused for enhanced surveillance and tailored care to optimize long term respiratory outcomes in childhood and beyond.

